# Tularemia seroprevalence in humans in the region of the Hittite-Arzawa War (Inner Aegean Region), where the first biological weapon was used 3300 years ago

**DOI:** 10.55730/1300-0144.5586

**Published:** 2022-11-03

**Authors:** İsmail DAVARCI, Canan ERYILDIZ, Duygu PERÇİN RENDERS, Ufuk BERBEROĞLU, Şaban GÜRCAN

**Affiliations:** 1 Department of Medical Microbiology, Faculty of Medicine, Trakya University, Edirne, Turkey; 2 Department of Medical Microbiology, Faculty of Medicine, Kütahya Health Sciences University, Kütahya, Turkey; 3 Department of Public Health, Faculty of Medicine, Uşak University, Uşak, Turkey

**Keywords:** Biological warfare agents, *Francisella tularensis*, seroepidemiological studies, tularemia, Turkey

## Abstract

**Background/aim:**

According to Egyptian records, tularemia emerged in the Canaan region, where it was first identified and spread to Anatolia over the Euphrates. It was used as an active biological weapon for the first time in the Hittite-Arzawa War in 1320–1318 BC.

This study aimed to investigate the seroprevalence of tularemia in the Inner Aegean Region, which is thought to be the region where this war was fought 3300 years ago.

**Materials and methods:**

Tularemia seropositivity in humans was investigated in 27 villages/neighborhoods in 3 districts in each of Manisa, Kütahya, and Uşak provinces. Before the study, the participants were informed about the disease via posters, and their blood samples were taken following filling out the questionnaire. Microagglutination tests were performed using in-house tularemia antigen and V plate for serological experiments. Rose-Bengal test was also performed on seropositive sera.

**Results:**

Of the total of 410 people, 226 (55.12%) were male. The mean age of the volunteers was 43.72 years. The highest participation was from Kütahya Province. According to the results of the tularemia microagglutination test, seropositivity was detected in 6 cases. It was determined that all of the seropositive volunteers were in Kütahya. When the tularemia antibody titers were examined, seropositivity was determined at 1/20–1/160 titers. No positivity was detected in the Rose-Bengal test for cross-reaction.

**Conclusion:**

Kütahya has been identified as a risky region in terms of tularemia in the Inner Aegean Region. In order to use the resources in the country economically, first of all, the risk areas in terms of tularemia should be determined by serological studies in all regions. In order to increase awareness about the disease, physicians and filiation teams should be trained in risky areas. Surveillance studies should be conducted to identify and monitor possible sources in areas identified as risky.

## 1. Introduction

*Francisella tularensis* is a Gram-negative aerobic bacillus and is the causative agent of tularemia. It was discovered by Edward Francis and named after Tulare County, where it was discovered. Tularemia, also known as rabbit fever or hunter’s disease, is a disease mostly seen in rural areas. Transmission occurs through gastrointestinal system, respiratory system, or direct skin contact with rabbits, rodents, and blood-sucking arthropods. Infection can occur in typhoidal, pneumonic, oculoglandular, oropharyngeal, ulceroglandular, and glandular forms. Although the incubation period varies between 1 and 21 days, it usually lasts 3–5 days. Symptoms vary according to the form of the infection [1].

According to Egyptian records, tularemia emerged in the Canaan Region (Lebanon, Syria, Jordan, and Israel), where it was first identified and spread to Anatolia over the Euphrates. It reached Western Anatolia in the 14th century BC through trade routes and wars. Arzawans settled in Western Anatolia attacked the Hittites in 1320 BC. However, the Hittites won the war in a short time, like two years. It is thought that the use of tularemia as a biological weapon played an important role in winning this war. By sending rams with tularemia to the enemy in biological warfare, the spread of the disease and the weakening of the other side were ensured [2].

Arzawa is a state founded in Western Anatolia at the beginning of the 2nd millennium BC; its first capital was Ephesus, and then the city of Zippasla in the east of Manisa. Arzawa, who initially accepted the sovereignty of the Hittites, declared sovereignty with the decrease of the power of the Hittites. It is thought that the root of Lydia, which was founded 500 years after the Hittites, goes back to the people of Arzawa. Uşak and Kütahya, which are on the Hittite-Arzawa border, and Manisa, which are close to this borderline, are cities of Arzawa [3–5]. The Hittite-Arzawa War probably took place in this region in 1320–1318 BC.

According to the literature and unpublished data of the Ministry of Health, tularemia has been reported in all provinces of the Aegean Region except Aydın [6–8]. However, no regional seroprevalence research has been conducted in the Aegean Region to date. This study aimed to investigate the seroprevalence of tularemia in the Inner Aegean Region, a possible region of the Hittite-Arzawa War, where tularemia was used as a biological weapon for the first time in history 3300 years ago.

## 2. Materials and methods

This study was carried out with the approval of the Trakya University Scientific Research Ethics Committee dated 13.04.2020 and numbered TUTF-BAEK 2019-381 and the support of the Trakya University Scientific Research Projects Commission (TUBAP) project numbered 2020-121.

For the study, three districts from each of 3 provinces in the Inner Aegean Region (Manisa, Kütahya, and Uşak provinces with high and low-risk areas dealing with agriculture and animal husbandry) were selected, and a sample was created by selecting three neighborhoods/villages from each district (Figure). In selecting the study regions, the places where tularemia cases were reported to the Ministry of Health were prioritized, and if there was no case report, a random selection was made. According to the 2019 data of the Turkish Statistical Institute, the population size consists of 38,710 people. Based on these data, the sample size was calculated as 410 people, as in the seroprevalence study previously conducted in the Thrace Region with a frequency of 1% and a confidence interval of 95% [9]. These people were tried to be distributed to be appropriate for the age groups that make up the population.

The independent variables of the cross-sectional study were determined as age, sex, residence, occupation, history of contact with game animals, history of eating and preparation of game meat, source of drinking and utility water, presence of mice in the environment, contact with mice, collecting food from the environment, feeding animals in and around the home, and occupation in nature. Dependent variables were determined as the result of microagglutination.

First, 27 villages/neighborhoods belonging to 9 districts planned to participate in the study were visited, and health workers and headmen of related villages were informed. Afterward, health workers and village people were informed about tularemia, its transmission routes, symptoms, disease prevention, and treatment methods. Information posters prepared about the disease were hung in places to be seen. Following this, the Tularemia Questionnaire Form was filled, and blood was taken from the volunteers included in the study in July 2021. The sera of the collected blood were separated and stored at + 4 °C until the day of the study within the same week.

Microagglutination tests were performed using patented *Francisella tularensis* antigen (patent no: TPE-2008/01623 B-ŞG). Saline was used as negative control and serum from patients with tularemia as the positive control. Rose-Bengal test was performed to evaluate cross-reactions for sera with positive tularemia microagglutination test.

Obtained data were evaluated with SPSS 22.0 program, and descriptive statistics were evaluated using Student’s *t*-test and chi-square test. When the *p*-value was < 0.05, it was considered statistically significant.

## 3. Results

The study group consisted of 410 people. The mean age was 43.72 ± 20.74 (2–89) years. The proportion of men was higher (men 53.17%). Those with social security were determined as 82.2%. Of women, 54.2% were housewives, and 26.1% of men were farmers. The average residence time in the region was 33.88 ± 24.11. Of people, 69.8% use only mains water, 20.7% use mains and treatment water. Of the study group, 23.2% stated that the mains water changed color on rainy days. Only 4.9% stated that they ate game animals. Nearly half (42.2%) were bitten by mosquitoes, 7.4% by other insects. Of the study group, 27.3% ate foods collected from nature (mushrooms, blackberries, wild strawberries, etc.), 12% kept animals at home, and 24.6% in the garden. Of the study group, 74.9% worked in nature, 28% had traveled elsewhere and among the age groups who volunteered to participate in the study, 61 years and over had the most participation (Table 1).

According to the tularemia microagglutination test results, no seropositivity was detected in Manisa and Uşak, while detected in 6 cases (1.46%) in the neighborhoods of central and Gediz districts of Kütahya. When the tularemia antibody titers were examined, seropositivity was determined at 1/20–1/160 titers (Table 2). No positivity was detected in the Rose-Bengal test for cross-reaction.

The mean age of the patients with seropositivity (65.2 ± 9.2) was higher than that of seronegative people (43.4 ± 20.7) (*t*-test, *p* = 0.01). In addition, those who were seropositive had a history of tuberculosis, and the characteristics of feeding animals in the garden were significantly different from those of the seronegative group (chi-square test, *p* = 0.002). When seropositive people were compared with seronegative ones in their neighborhood, there was no difference in their exposure to risk factors. However, when the neighborhoods where seropositives live are compared with the neighborhoods/villages where seronegatives live, in terms of exposure to risk factors, activities in nature such as collecting mushrooms and blackberries from the environment (*p* = 0.003) and working in the field and garden (*p* = 0.001) were higher.

## 4. Discussion

Ticks play an important role as a reservoir and vector in Tularemia, as *Francisella tularensis* can transmit transovarial and transstadial. The causative bacteria are transmitted to humans and other mammals through ticks. In addition, agents have been detected in many living things such as birds, fish, and insects [10]. For these reasons, when the agent and disease are detected in a region, it exists for many years [11]. *Francisella tularensis* may have continued by settling in these regions after being used as a biological weapon for the first time 3300 years ago in the study area. When we look at the unpublished data from the Ministry of Health, the fact that there were 28 human case reports in Kütahya, 3 in Uşak, and 2 human cases in Manisa between 2011 and 2018 and the detection of other seropositive cases in 2021 in the presented study is evidence that the agent continues to exist in this region.

The first tularemia cases in Turkey were reported from the Thrace Region (European Part of Turkey). Tularemia outbreaks have been detected in villages of Tekirdağ and the Lüleburgaz district and some villages along with the Kaynarca Stream in Kırklareli [10]. In the same region, outbreaks were described in the following years [11,12]. The first regional study in Turkey on tularemia seroprevalence was also conducted in the Thrace Region [9]. Although there are case reports from almost all provinces in the Aegean Region, no regional seroprevalence studies have been encountered [6–8]. Within the framework of the Turkey Zoonotic Diseases Action Plan (2019–2023), it is aimed to put the first step of the field research model into practice by carrying an exemplary study out following the aim of systematically revealing the current situation in the country and planning control measures with the data to be obtained [13].

Although tularemia outbreaks were reported in the Thrace Region in 1936 and 1945, no cases of tularemia were reported for a long time in the following years. In the seroprevalence study, which was planned to reveal the reason for this situation, seropositive cases were detected in some villages. This fact was interpreted as cases with tularemia, but it could not be diagnosed because it was not considered in the differential diagnosis [9]. Indeed, following this study, tularemia outbreaks in Edirne and Tekirdağ villages were detected after 60 years [11,12]. In subsequent studies, vectors such as mice, stream water, tap water used in homes, and incidence studies in humans were closely followed in terms of tularemia in the region [14,15]. In these surveillance studies, the causative agent of tularemia was detected first in mice and then in mains water in nearby areas by molecular methods. The health authorities took the necessary actions to prevent a potential tularemia epidemic before it occurred, for the first time in Turkey [14,16]. It was hoped that this study, carried out in the Inner Aegean Region, would raise awareness, identify priority areas for further research, and enable early warning opportunities.

In the present study, in the regions where seropositive cases live, collecting food such as mushrooms and blackberries from the environment and performing activities in nature such as working in the field and garden can be considered factors that increase the risk of tularemia. In order to reduce this risk, it was thought that it would be beneficial to comply with hygienic rules in the collection and eating of food in nature, use chlorinated drinking water, and take measures to protect against ticks and insects during activities in nature.

*Francisella tularensis* is a factor that has a critical role as a biological terror agent [10]. It is stated in the Egyptian records that the causative bacterium was used in the Hittite-Arzawa War as the first biological terror attack [2,17]. This war probably took place in the Inner Aegean Region, where the provinces of Uşak, Kütahya, and Manisa are located in the study area. This study also has historical importance because information about the current state of tularemia in the region, which was used as the first biological warfare tool 3300 years ago, was obtained. According to the presented study results, it was determined that tularemia continues to exist in these regions.

Half of the six seropositive cases were detected in the Arslanlı neighborhood of the Centrum district in the province of Kütahya, and the highest antibody titers were also in Arslanlı suggested that the tularemia risk of this region is very high. According to unpublished data from the Ministry of Health, the fact that 6 of 28 cases reported from Kütahya between 2011 and 2019 were reported from Arslanlı and that this neighborhood is the place where the highest number of tularemia cases reported in Kütahya is evaluated as data emphasizing the magnitude of this risk. In the study presented in the Eskiureyil neighborhood of the Centrum district of Kütahya, in response to 2 case reports in 2011, seropositivity was detected in 1 volunteer at 1/80 titer. Moreover, it is remarkable that seropositivity was detected in 2 volunteers at 1/20 and 1/40 titers in the Pulat neighborhood in Gediz district, where 1 case was reported in 2011. According to unpublished data from the Ministry of Health, there were 3 case reports in Uşak and 2 cases in Manisa between 2011 and 2019. In the light of these data, the fact that all six seropositive cases were in Kütahya was evaluated as another piece of data confirming that the risk is higher in Kütahya compared to other provinces included in the study. It would be beneficial to closely investigate tularemia in humans, animals, water, and other natural elements through surveillance and follow-up in such risky areas. Thanks to tularemia surveillance in risky areas in the Thrace Region, the risks of tularemia outbreaks could be noticed earlier, and outbreaks could be prevented with early measures [14,16]. For these reasons, tularemia surveillance in humans, animals, waters, and nature, especially in Kütahya Province, can provide early detection of possible outbreaks.

According to unpublished data from the Ministry of Health, the names and surnames of people affected by the outbreak in previous years were evaluated with the seropositive cases in our study. As a result of this evaluation, it was determined that none of the seropositive cases in our study had the same name in the previous report of tularemia, but only one seropositive case had a surname similar to a patient in the same locality. These data showed that tularemia cases might be more numerous than those diagnosed in Kütahya. It is difficult to say whether these cases are new or formerly affected by these diseases. In any case, it can be thought that physicians do not sufficiently bring these diseases to mind in the differential diagnosis and that awareness about the disease is lacking. For this reason, it is thought that it would be beneficial to provide training to increase physicians’ awareness about tularemia, especially in risky areas.

Following the detection of index cases in tularemia outbreaks in Turkey, serological studies performed to determine the extent of the outbreak in the region where the patient is thought to have acquired the causative agent also ensure the detection of asymptomatic seropositive cases. In tularemia outbreaks in Turkey, only less than 14% of seropositive cases are asymptomatic. All other seropositive cases present as symptomatic. In addition, conducting studies to determine the source during outbreaks plays a critical role in controlling the outbreak and preventing new cases [11,16,18]. Filiation studies can reveal all these findings. The fact that seropositive cases generally do not have surnames similar to those affected by the disease in the past suggests that there was not enough research done on other people outside the family during the disease. Accordingly, it brings to mind that the filiation studies could not be carried out healthily. For this reason, it has been beneficial to train the filiation teams to carry out studies that will reveal the epidemic in all its dimensions.

Tularemia cases in Turkey are usually water-borne and occur in the form of outbreaks [10]. It is crucial to carry out filiation studies after the outbreaks occur, but it is more critical to prevent them. This can only be possible with preepidemic surveillance studies [16]. As a result, to use the resources in the country economically, as in this study, the risk areas should be determined by serological studies in all regions, and then surveillance studies should be carried out to identify and follow up the possible sources in these regions. According to the results of these studies, the precautions and activities to be taken regarding the diseases should be determined.

## Figures and Tables

**Figure:**
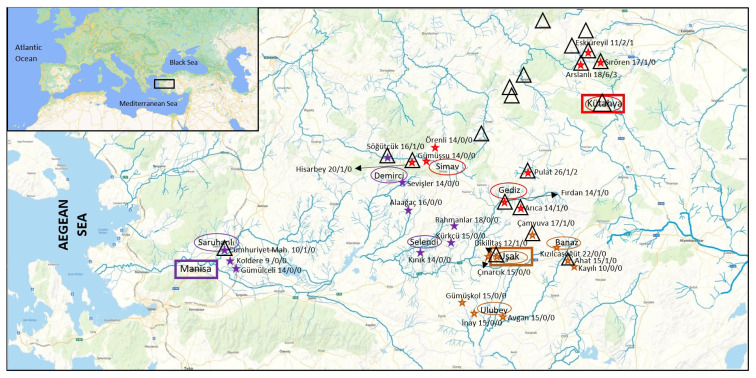
Study areas and their features. ★ Villages-neighborhoods where samples were taken and number of volunteers/number of cases before 2019/number of seropositive cases in 2021. Places where tularemia cases were reported before 2019 ▵ Provinces included in the study ⬭ Districts included in the study 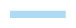
 Stream/creek

**Table 1:** Distribution of those who volunteered to participate in the study by age groups and sex.

Age groups	Male	Female	Total (%)
**1–10**	19	11	30 (7.32)
**11–20**	31	26	57 (13.90)
**21–30**	14	16	30 (7.32)
**31–40**	13	34	47 (11.46)
**41–50**	44	34	78 (19.02)
**51–60**	38	22	60 (14.63)
**61 and over**	67	41	108 (26.34)
**Total (%)**	**226** (**55.12**)	**184** (**44.88**)	**410** (**100**)

**Table 2:** Characteristics of cases with seropositivity in tularemia microagglutination test.

Case number	Province	District	Village/neighborhood	Age	Sex	Profession	Antibody titer
**1**	Kütahya	Centrum	Arslanlı	70	Female	Housewife	1/160 +
**2**	Kütahya	Centrum	Arslanlı	68	Female	Housewife	1/80 +
**3**	Kütahya	Centrum	Arslanlı	65	Female	Housewife	1/80 +
**4**	Kütahya	Centrum	Eskiüreyil	47	Female	Housewife	1/80 +
**5**	Kütahya	Gediz	Pulat	70	Female	Housewife	1/40 +
**6**	Kütahya	Gediz	Pulat	71	Male	Retired miner	1/20 +
